# Could consumption of yam (*Dioscorea*) or its extract be beneficial in controlling glycaemia: a systematic review

**DOI:** 10.1017/S0007114521003706

**Published:** 2022-08-28

**Authors:** Waad Z. Alharazi, Anthony McGowen, Peter Rose, Preeti H. Jethwa

**Affiliations:** Division of Food, Nutrition and Dietetics, School of Biosciences, University of Nottingham, Sutton Bonington Campus, Loughborough LE12 5RD, UK

**Keywords:** Yam, Dioscorea, Type II diabetes, High fat diet, Glycaemia, Rodent

## Abstract

Yam (*Dioscorea spp.*) and its associated extracts have been shown to possess a variety of biological activities and identified as beneficial in the control of glycaemia in patients with type II diabetes mellitus (T2DM). The objective was to conduct a systematic search of the literature to investigate whether yam and its extract can improve glycaemia and whether the consumption of yam could be beneficial for managing T2DM. Using the Preferred Reporting Items for Systematic Reviews and Meta-Analyses guidelines and the Population, Invention, Comparison and Outcome framework, three databases (PubMed, Scopus and Web of Science) were searched using a key term strategy. Strict inclusion criteria were employed to identify all relevant and available studies. The quality of these studies was assessed using SYRCLE’s Risk of Bias tool. Ten studies were included, and all studies consisted of findings from rodent models of diabetes, including animals consuming high fat diets or genetic models of diabetes. All ten studies showed that the consumption of yam and/or its extracts (containing dioscin, dioscorin, diosgenin, DA-9801/02 or Chinese yam polysaccharides) improved glycaemia. These included improvements in fasting blood glucose and reductions in glucose and increase in insulin levels following a glucose tolerance test. Furthermore, significant changes in body weight and adiposity were observed in nine studies, these included improvements in lipid biomarkers in four and reductions in inflammatory markers in one. The current work indicates that the consumption of yam or its extracts can be beneficial for improving blood glucose; however, the molecular mechanism for these effects remains largely unknown. Future trials on human subjects are warranted.

Diabetes mellitus is a non-communicable metabolic disease projected to affect 366 million by 2030^(1–3)^. The most common form of diabetes is type II diabetes mellitus (T2DM) which often begins with obesity associated with insulin resistance and glucose tolerance leading to hyperglycaemia, impaired *β*-cell function and a decrease in insulin secretion^(4)^. Furthermore, impairment in lipid and lipoprotein metabolism, increase in oxidative stress, diminished vascular endothelial function and high blood pressure are also common in T2DM^(5,6)^. Chronic exposure to these complications often leads to health conditions including peripheral neuropathy, retinopathy and nephropathy alongside an increased mortality rate^(7)^.

Therefore, controlling blood glucose is important to prevent diabetic complications and to improve health of patients. While a number of hypoglycaemic agents have been developed, based on current understanding of the pathophysiology of T2DM, their use results in a myriad of side effects and the initial improvements in glycaemia are usually not sustained^(8,9)^.

Obesity is a risk factor for the development of T2DM, and dietary management is thought to reduce the burden on islet cells and thus improve glucose levels, inflammation and lipid profile^(10)^. Recent evidence suggests that the regular consumption of foods with bioactive compounds may benefit health related to prevention or management of chronic diseases^(11–13)^.

Yam (*Dioscorea*), an angiosperm (flowering plant) not botanically related to sweet potato (*Ipomoea*), is commonly consumed in the Asian and African continents^(14)^. In the African populations, the prevalence of T2DM ranges from 3·5 % in rural area to 7·5 % in urban area^(15,16)^. In Asia, yam has been used in traditional Chinese medicine as a natural medicine for T2DM^(17)^.

Of particular interest in this region are the numerous extracts, which include allantoin, dioscorin, sapogenins, prosapogenin, gracillin, choline, l-arginine, polysaccharides and proteins. Several *in vitro* and *in vivo* studies have highlighted the anti-diabetic action of a number of these extracts, including dioscorea ethanol extract^(18)^, total saponins^(19–22)^ allantoin^(23,24)^, water soluble polysaccharides^(25)^, DA-9801^(26)^ and diosgenin^(27,28)^. However, many of these studies have been conducted in animal models in which diabetes was induced by streptozotocin (STZ)^(19–22)^ or alloxan^(18,25)^. These are popular methods but induce hyperglycaemia via the destruction of the pancreatic islets and do not mimic the insulin resistance presented in human patients with T2DM^(29)^.

Therefore, we conducted a systematic review to search the literature to investigate whether yam and its extract can improve glycaemia in diet-induced and spontaneous T2DM *in vivo* models and determine whether the consumption of these could be a diet modification.

## Method

The review was constructed in accordance with the Preferred Reporting Items for Systematic Reviews and Meta-Analyses guidelines^(30)^.

### Searching strategy

A computerised search of the literature was performed using three databases (PubMed, Scopus and Web of Science) between April 2020 and June 2020. The searching process followed the Population, Invention, Comparison and Outcome framework. The population was T2DM animal models or human patients diagnosed with T2DM, the intervention was yam or yam extracts in comparison with controls who do not receive the intervention and measuring the outcome is the effect of the yam intervention on complications associated with T2DM such as insulin sensitivity and glucose tolerance. Search of terms was conducted through the literature to define the keywords: yam OR ‘yam extract’ OR *Dioscorea* AND diabetes OR antidiabet × OR glycaem × OR insulin OR glucose OR T2DM. Two independent reviewers (WA and AM) assessed the titles, abstracts and full articles, based on strict inclusion and exclusion criteria; any disagreements with the section of the article were resolved through discussion. Full articles of the selected titles were retrieved, and the reference lists of these were searched to identify any additional publications.

#### Selected studies criteria

All related articles from inception were considered as there have been no previous systematic reviews conducted to investigate the relationship of yam and its phytochemicals to the anti-diabetic effects identified during our search.

#### Inclusion criteria

- Only articles written in English were eligible to avoid any misleading translations.
- All studies must have described either animal models with diet induced T2DM or human participants who have been diagnosed with T2DM by a medical profession.
- Any yam and yam extracts were considered.
- All studies must have a measure of glycaemic parameters.
- Randomised clinical trials.
- Fully published studies.

#### Exclusion criteria

- Chemically induced hyperglycaemia using pharmaceutical agents (e.g. STZ).
- Non-diabetic model.
- *In vitro* cell studies exploring the cellular mechanisms.
- Systematic reviews or critical reviews.
- Traditional Chinese medicine or any traditional medicine that contains other plants in addition to or alternative to yam.

### Measured outcomes

The primary outcomes of this review are the effect on glycaemic parameters such as fasting blood glucose (FBG), HbA_1_c, glucose tolerance test (GTT), insulin levels, homoeostatic model assessment of insulin resistance (HOMA-IR), insulin-glucose ratio, insulin sensitivity index, insulin tolerance test (ITT), metabolic clearance rate (MCR) and adiposity insulin resistance index, while secondary measurements were factors associated with glycaemic control. These include body weight, lipid profile (total fat, white adipose tissue, total cholesterol (TC), TAG, LDL and HDL and NEFA), blood pressure (systolic blood pressure (SBP) and diastolic blood pressure (DBP)) and inflammatory parameters (leptin, IL-1*β*, IL-10, matrix metalloproteinase (MMP), NF-κB).

### Data extraction

A standard data extraction form was used to obtain data from the studies and charted using Excel (Microsoft Excel). Data extracted included title, author, publication year, country, study population, sample size, diabetic model, exposure to yam genus or yam extracts and outcomes (FBG, HbA_1_c, glucose levels following GTT, insulin levels, HOMA-IR, MCR and adiposity insulin resistance index), body weight change, lipid profiles (total fat, white adipose tissue, TC, TAG, LDL and HDL and NEFA), blood pressure (SBP and DBP) and inflammatory parameters (leptin, IL-1*β*, IL-10, MMP, NF-κB)). The results from each study alongside statistical outcomes were also extracted.

### Data analysis

The relevant results were expressed in tables. The key characteristics of the selected papers included the study design, population, model used, number of the sample, outcome measures and doses of intervention groups. The significant effects in response to the intervention were charted to compare across the article retrieved. Raw values for the primary outcome measures were not reported consistently across all studies; therefore, we were unable to compare the magnitude of the effect on the primary outcome measures and conduct a meta-analysis.

### Quality assessment

The SYRCLE’s Risk of Bias tool was used to assess quality assessment due to the lack of human participant studies. This is an adapted version of the Cochrane Risk of Bias tool^(31)^ consisting of ten items relating to six types of bias. Items 1, 3, 8, 9 and 10 are adopted from the Cochrane Risk of Bias tool, while items 2, 4, 5, 6 and 7 have been adapted or replaced to allow for appropriate assessment of animal studies^(32)^ (online Supplementary Table S1). Signalling criteria were used to determine and assign a judgement of low, high or unclear risk of bias.

The quality assessment examined multiple types of bias: selection, performance and direction, attrition and reporting. Selection bias (items 1, 2 and3) was assessed by sequence generation, baseline characteristics and allocation concealment. Performance bias (items 4 and 5) was assessed by randomised housing and blinding relating to researchers and/or animal caregivers. Detection bias (items 6 and 7) assessed any random outcome assessment and blinding as it can lead to multiple types of bias. Attrition bias (item 8) was explored by assessing incomplete outcome data, while reporting bias (item 9) assessed selective outcome reporting. Other sources of bias were covered by item 10 (online Supplementary Table S1).

## Results

### Eligibility of studies

**Fig. 1.**
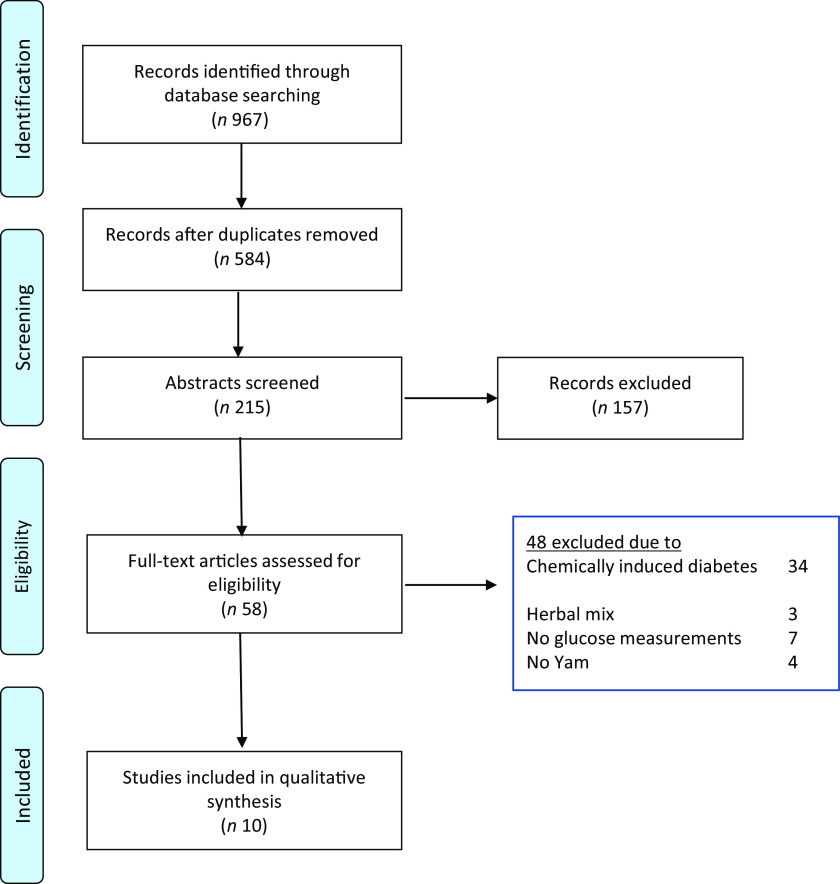
Flow diagram demonstrating the identification and selection of relevant research (PRIMSA, 2015).

Using three electronic databases (PubMed/Medline, Scopus and WoS), we identified 967 papers published between 1962 and April 2020. Following the removal of duplicates and an initial title screen, 215 studies were assessed in more detail. Of these, fifty-eight were evaluated against stringent inclusion/exclusion criteria, thirty-four used medically induced diabetic models, seven utilised a mixture of compounds which contained extracts from sources other than yam, seven did not measure glucose levels and four did not include yam. This left ten studies eligible for inclusion (Fig. 1).

### Quality assessment

All ten papers had ‘Fair’ as a final judgement for quality (Table 1 and online Supplementary S1). Based on the assessed bias criteria, the only study that scored ‘High Risk’ at the ‘Selection Bias’ questions 1, 2, 3 was ref. [33]. While ‘Performance and Detection bias’ questions 4 and 6 had unclear and low risk in all studies, questions 5 and 7 which assess the blinding for caregiver and investigators were judged as ‘High Risk’; however, this may not affect the overall judgement where ref. [34] found in their meta-analysis that blinding in animal trials is not statistically significant. In regard to ‘Attrition Bias’, question 8 highlighted two papers as ‘High Risk’ which are ref. [35] where eight out of ten mice outcomes data were reported and ref. [36] where eight out of fourteen mice outcomes data were reported.

**Table 1.** SYCRLE tool for Risk of Bias (RoB) of selected studies

	Random sequence generation	Baseline characteristics	Allocation concealment	Random housing	Blinding – caretakers	Random outcome assessment	Blinding – assessors	Incomplete outcome data	Selective outcome reporting	Other bias	Rating
Xu *et al.*, 2020	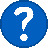	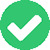	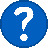	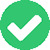	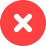	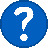	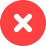	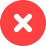	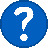	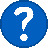	Fair
Cheng *et al.*, 2019	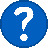	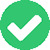	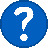	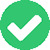	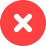	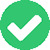	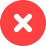	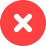	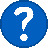	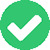	Fair
Li *et al.*, 2019	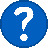	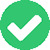	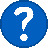	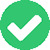	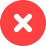	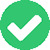	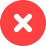	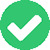	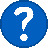	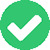	Fair
Wu *et al.*, 2018	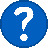	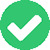	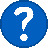	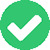	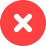	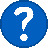	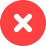	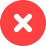	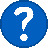	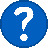	Fair
Hashidume *et al.*, 2018	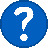	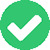	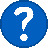	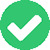	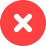	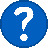	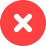	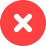	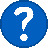	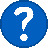	Fair
Sato *et al.*, 2017	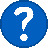	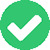	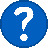	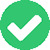	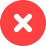	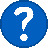	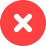	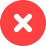	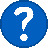	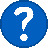	Fair
Shih *et al.*, 2015	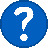	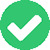	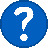	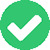	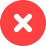	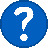	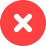	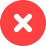	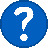	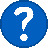	Fair
Moon *et al.*, 2014	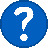	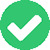	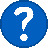	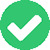	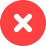	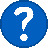	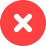	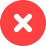	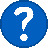	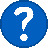	Fair
Kim *et al.*, 2012	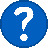	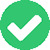	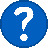	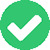	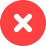	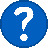	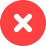	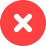	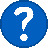	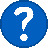	Fair
Hsu *et al.*, 2007	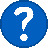	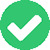	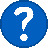	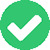	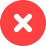	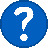	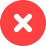	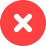	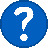	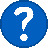	Fair

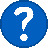
, unclear risk of bias, 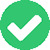, low risk of bias, 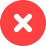, high risk of bias, according to SYCRLE recommendations.

### Study characteristics

The characteristics of the studies are summarised in Table 2. The selected studies were located in Asian countries, though no restraints were placed on location. The experimental length varied from 4 weeks to 19 weeks with an average of 11 weeks. No studies included human participant diagnosed with T2DM, and all studies were carried out in rodent model, although two studies utilised *in vitro* models as part of their study design^(33,35)^. In the rodent model, T2DM was induced by consuming high fat diet (HFD) in rats or mice, the KK-A^y^ mice, which spontaneously exhibit T2DM and the *db/db* mice, a genetic model of T2DM, were fed HFD, while the OLETF mice fed normal diet; one study, ref. [37] fed the rodent model rats with high fructose diet to induce hyperglycaemia. The studies utilised a variety of *Dioscorea* species although two studies did not mention the yam species^(38,39)^. A variety of yam extracts were used in nine of the studies; these included components, dioscin^(35,38)^, dioscorin^(39,40)^, diosgenin^(41)^, DA-9801 and DA-9802^(33,42)^ and *Dioscorea esculenta* powder^(43)^, while one study used raw material of *Dioscorea Opposita* (a synonym of two species of yam Dioscorea polystachya and Dioscorea oppositifolia)^(37)^. The yam and/or its extracts were obtained via various methods; five articles reported their extraction methods from raw yam while two articles purchased the yam from external sources^(33,35–37,40–42)^. The extraction methods included aqueous ethanol extraction from dried yam^(33,35,42)^, water extraction and alcohol precipitation method and raw flush sample mixed with Tris buffer and purified with DE-52 ion exchange chromatography^(36,40)^. These were administered either orally or with saline through gavage at varying doses ranging from 5 to 100 mg/kg. Biochemical measurements included measurements of glycaemia (FBG, GTT, HbA1c, HOMA-IR, insulin-glucose ratio, insulin sensitivity index, ITT, MCR and adiposity insulin resistance index), lipid profile (total fat, white adipose tissue, TC, TAG, LDL and HDL and NEFA), blood pressure (SBP and DBP) and inflammatory markers (leptin, IL-1*β*, IL-10, MMP, NF-κB; Tables 3 and 4).

**Table 2.** Key characteristics of the selected studies

Article	Country of origin	Study design	Animal model		Yam species	Yam/its extract	Mode of administration	Study duration	Biological parameter	Extraction method
			Diabetic	Control						
Xu *et al.*, 2020	China	RCT	HFD KK-A^y^ mice	HFD KK-A^y^ mice	D. nipponica rhizomes	Dioscin	Gavage – OD/8 weeks	8 weeks	– FBG, OGTT, HbA_1_c	– 50–60 % aqueous ethanol
									– IL, HOMA-IR, ISI, ITT	
									– weight	
									– TC, TAG, LDL, HDL, NEFA	
Cheng *et al.*, 2019	China	RCT	HFD C57BL/6 mice	HFD C57BL/6 mice	Chinese yam	CYP	Orally – OD/8 weeks	14 weeks	– FBG	– Alcohol extraction using water bath
									– ITT	
									– Weight	– DdH_2_O used for washing
									– TC, TAG, LDL, HDL	
Li *et al.*, 2019	China	RCT	HFD C57BL/6 mice	HFD C57BL/6 mice		Dioscin	Gavage – for 12 weeks	12 weeks	– FBG	– Not reported
									– IL, HOMA-IR, Adipo-IRI	
									– weight	
									– TC, TAG, NEFA	
									– TF, adiposity index	
Wu *et al.*, 2018	Taiwan	RCT	HFD C57BL/6 mice	HFD C57BL/6 mice	D. alata	Dioscorin	Gavage – OD/19 weeks	19 weeks	– OGTT	– Homogenised with Tris buffer
									– Weight	– Eluted with NaCl and dialysed with deionised water
									– TC, TAG, LDL	
Hashidume *et al.*, 2018A	Japan	RCT	HFD KK-A^y^ mice	HFD KK-A^y^ mice	D. batatas	Sanyaku Diosgenin	Orally – for 11 weeks	11 weeks	– FBG	– Diosgenin purchased from Sigma Chemical
									– Weight	– Sanyaku purchased in freeze-dried powder form
									– TAG	
									– WAT	
Sato *et al.*, 2017	Japan	RCT	OLEFT mice	LETO mice	D. esculenta	Diosgenin	Added to diet with *ad libitum* access	8 weeks	– FBG	– Not reported
									– MCR	
									– Weight	
Shih *et al.*, 2015	Taiwan	RCT	HFD MW rats	HFD MW rats		Dioscorin	Orally – OD/5 weeks	10 weeks	– OGTT	– Not reported
									– Weight	
									– SBP/DBP	
Moon *et al.*, 2014	Korea	RCT	HFD *db/db* mice (C57BLKS/J)	HFD *db/db* mice (C57BLKS/J)	D. japonica & D. nipponica	DA-9081	Orally – OD/12 weeks	12 weeks	– FBG	– 50 % aqueous ethanol
									– Weight	– At room temperature for 48 h
										– Filtered and concentrated
Kim *et al.*, 2012	Korea	RCT	HFD C57BL/6 mice	HFD C57BL/6 mice	D. batatas	DA-9082	Gavage – OD/7 weeks	12 weeks	– FBG, OGTT	– Water-ethanol solution for 24 h
									– IL, IGR	– Filtered and concentrated
									– Weight	
Hsu *et al.*, 2007	Taiwan	RCT	High fructose diet MW rats	MW rats	D. opposite thumb	D. opposite thumb	Orally through water	4 weeks	– FBG, IPTT, HbA_1_c	– Purchased from Shen Chang Pharmaceutical Co., Ltd

RCT, randomised control trial; HFD, high fat diet; MW, male Wister; D., Dioscorea; CYP, Chinese yam polysaccharides; OD, once daily; FBG, fasting blood glucose; OGTT, oral glucose tolerance test; HbA1C, glycated haemoglobin; IPTT, intraperitoneal glucose tolerance test; IL, insulin level; HOMA-IR, homoeostatic model assessment for insulin resistance; ISI, insulin sensitivity index; ITT, insulin tolerance test; IGR, insulin-glucose ratio; MCR, metabolic clearance rate; Adipo-IRI, adiposity insulin resistance test; TC, total cholesterol; TF, total fat; WAT, white adiposity tissue; SBP, systolic blood pressure; DBP, diastolic blood pressure; DdH_2_O, double distilled water.

**Table 3.** Effects of yam consumption on glycaemic parameters measured in the selected studies

Article	Yam/extract	Model	Dose	Glucose		HbA_1_c (%)	Insulin	IGR	HOMA-IR	ISI	MCR	ITT	Adipo-IRI
				FBG	GTT								
Xu *et al.*, 2020	Dioscin	HFD KK-A^y^ mice	20 mg/kg	NS	0–120 min ↓**	↓**	↓**		↓**	↓**		↓**	
			40 mg/kg	↓**		↓**	↓**		↓**	↓**		↓**	
			80 mg/kg	↓**		↓**	↓**		↓**	↓**		↓**	
Li *et al.*, 2019		HFD mice	5 mg/kg	NS			NS		NS				↓*
			10 mg/kg	↓*			↓*		↓*				↓**
			20 mg/kg	↓*			↓**		↓*				↓**
Wu *et al.*, 2018	Dioscorin	HFD mice	80 mg/kg		30 min ↓**								
					60 min ↓**								
					90 min ↓**								
					120 min ↓*								
Shih *et al.*, 2015		HFD rats	50 mg/kg		0–120 min ↓*								
Moon *et al.*, 2014	DA-9801	*db/db* mice	30 mg/kg	↓*									
			100 mg/kg	↓*									
Kim *et al.*, 2012	DA-9802	HFD mice	100 mg/kg	↓*	0–5 min: NS 30–120 min ↓*		↓*	↓*					
Hsu *et al.*, 2007	D. Opposita Thunb.	FD rats	DHW	↓*	↓*	↓*							
			DHW/D.	NS	NS	NS							
			D. 4·2 mg/kg	↓*	↓*	↓*							
Cheng *et al.*, 2019	CYP	HFD mice	10 mg/kg	NS								NS	
			20 mg/kg	↓*								↓*	
Sato *et al.*, 2017	Diosgenin	ND OLEFT	0·3 % added to ND	6 weeks↓*							↑*		
		mice		8 weeks↓*									
Hashidume *et al.*, 2018	Sanyaku Diosgenin	HFD KK-A^y^ mice	0·05 % added to HFD	↓*									
				NS									

NS, not significant; HFD, high fat diet; FD, fructose diet; ND, normal diet; FBG, fasting blood glucose; GTT, glucose tolerance test; IGR, insulin-glucose ratio; ISI, insulin sensitivity index; MCR, metabolic clearance rate; ITT, insulin tolerance test; Adipo-IRI, adiposity insulin resistance index.

Arrows indicate direction of change;

* *P* < 0·05; **P < 0·01.

**Table 4.** Effects of yam consumption on parameters associated with T2DM measured in the selected studies

Article	Yam/extract	Model	Dose	Δ Wt.	Lipid profile					BP	Inflammation				
					TAG	TC	LDL	NEFA	HDL		Lpn	IL1*β*	IL10	MMP	NF-κB
Xu *et al.*, 2020	Dioscin	HFD KK-A^y^ mice	20 mg/kg	↓*	↓**	↓**	↓**	↓*	↑*						
			40 mg/kg	↓*	↓**	↓**	↓**	↓**	↑*						
			80 mg/kg	↓*	↓**	↓**	↓**	↓**	↑*						
Li *et al.*, 2019		HFD mice	5 mg/kg	NS	NS	NS		NS							
			10 mg/kg	↓*	↓*	↓*		NS							
			20 mg/kg	↓*	↓*	↓*		↓*							
Wu *et al.*, 2018	Dioscorin	HFD mice	80 mg/kg	↓*	NS	↓*	↓*								
Shih *et al.*, 2015		HFD rats	50 mg/kg	NS						SBP ↓*					
										DBP NS					
Moon *et al.*, 2014	DA-9801	*db/db* mice	30 mg/kg	NS											
			100 mg/kg	NS											
Kim *et al.*, 2012	DA-9802	HFD mice	100 mg/kg	NS											
Hsu *et al.*, 2007	D. Opposita Thunb.	FD rats	DHW												
			DHW/D.												
			D. 4·2 mg/kg												
Cheng *et al.*, 2019	CYP	HFD mice	10 mg/kg	NS	NS	NS	NS		↑*		NS	↓*	↓*	↓*	↓*
			20 mg/kg	↓*	NS	↓*	↓*		↑*		↓*	↓**	↓**	↓**	↓**
Sato *et al.*, 2017	Diosgenin	ND OLEFT	0·3 % added to ND	NS											
		mice													
Hashidume *et al.*, 2018	Sanyaku Diosgenin	HFD KK-A^y^ mice	0·05 % added to HFD	NS	NS										

NS, not significant; HFD, high fat diet; FD, fructose diet; ND, normal diet; ΔWT, change in body weight; TC, total cholesterol; BP, blood pressure; SBP, systolic blood pressure; DBP, diastolic blood pressure; Lpn, leptin; MMP, matrix metalloproteinases; NF-κB, nuclear factor kappa-light-chain-enhancer of activated B cells.

Arrows indicate direction of change;

* *P* < 0·05; **P < 0·01.

### Effects of yam extract on measurements of glycaemia

#### Fasting blood glucose/glucose tolerance test

All the included studies measured glucose and, although these were measured via a variety of methods (FBG/GTT), they all showed that treatment with either yam or its extract led to significant improvements in glucose tolerance compared with the controls (Table 2). FBG was reported in all studies except ref. [39,40], while GTT was measured in five of the ten studies^(35,37,39,40,42)^. Dioscin, dioscorin, DA-9801, DA-9802, diosgenin and CYP improved FBG (*P* < 0·05), while dioscin, dioscorin and DA-9802 were shown to improve GTT from 30 to 120 min after oral or intraperitoneal glucose load (*P* < 0·05). The lowest working doses of yam or yam extract ranged from 10 to 100 mg/kg in the HFD model, while in the genetic models of diabetes (KK-A^y^, *db/db*, OLEFT) the lowest working doses ranged from 0·5 to 30 mg/kg (Table 3).

#### HbA1c

HbA1c was measured in two of the ten studies; both showed that the consumption of yam or its extract significantly reduced HbA1c^(35,37)^. Xu *et al.* showed that dioscin reduced HbA1c in KK-A^y^ at all doses, including the lowest dose of 20 mg/kg, although this dose had no effect on FBG, while Hsu *et al.* showed that the consumption of *D. Opposita Thunb* had a significant reduction (*P* < 0·05) (Table 3)

#### Insulin

Fasting insulin levels were measured in three of the ten papers and all showed significant decrease in insulin in a dose-dependent manner (*P* < 0·05; Table 3)^(35,38,42)^. HFD mice treated with dioscin (10 and 20 mg/kg) or DA-9082 (100 mg/kg) reduced fasting insulin levels in HFD mice^(38,42)^. Dioscin also significantly reduced fasting insulin levels in KK-A^y^ mice at all three doses 20, 40 and 80 mg/kg^(35)^.

#### Homoeostatic model assessment of insulin resistance, insulin sensitivity index, insulin tolerance test, insulin-glucose ratio and metabolic clearance rate

HOMA-IR was measured in two of the ten studies, while insulin sensitivity index was measured in one, ITT was measured in two, insulin-glucose ratio was measured in one and MCR was measured in one (Table 3)^(35,36,38,42,43)^. Administration of the dioscin in HFD mice and KK-A^y^ significantly reduced HOMA-IR, insulin sensitivity index and glucose following an ITT at all three doses (20–80 mg/kg)^(35)^, HOMA-IR in ref. [38] reduced in 10, 20 mg/kg dioscin groups, while administration of diosgenin in OLEFT mice significantly increased MCR and DA-9802 in reduced insulin-glucose ratio significantly^(42,43)^. ITT in ref. [36] was significant in CYP 20 mg/kg.

Adiposity insulin resistance indexOne study measured adiposity insulin resistance index and showed that dioscin treatment (5–10 mg/kg) resulted in significant reduction (*P* < 0·05)^(38)^.

### Other factors related to glycaemic control

#### Body weight changes, total fats, white adiposity tissue and adiposity index

Nine of the ten studies measured body weight; of these, four observed a significant decrease in body weight regardless of diabetic model (HFD, KK-A^y^ or C57BL/6) or yam extract (dioscin, CYP, dioscorin; *P* < 0·05)^(35,36,38,40)^. Li *et al*. found that the decrease in body weight following dioscin treatment (10–20 mg/kg) was due to a 14 % decrease in total fat (*P* < 0·05). However, ref. [33,39,41–43] did not observe any changes in body weight nor changes in white adiposity tissue following treatment with sanyaku, diosgenin, dioscorin, DA-9801 and DA-9802 (Table 4).

#### Lipid profile (total cholesterol, TAG, LDL, HDL and NEFA)

Five studies measured the lipid profile biomarkers^(35,36,38,40,41)^, these included TC, TAG, LDL, HDL and NEFA (Table 4); of these, four found changes in some lipid biomarkers, only ref. [41] observed no differences between treatment and control. References [35] and [38] revealed significant reductions in TC, TAG, LDL and NEFA following dioscin and treatment (*P* < 0·05) in both KK-A^y^ and HFD diet mice. These results were partially supported by ref. [36,40]; while they observed significant reduction in TC and LDL following dioscorin and CYP treatment, respectively, in HFD model, no changes in TAG were observed. In addition, both refs [35,36] found that dioscin and CYP also increased HDL levels.

#### Blood pressure

SBP and DBP were measured in only one of ten studies. Reference [39] showed a significant decrease in SBP (*P* < 0·05), but not DBP (Table 4).

#### Inflammatory markers

One of the ten studies measured markers of inflammation and adipocytokines, these included leptin, IL1*β*, IL-10, MMP and NF-κB. Cheng et al. showed that administration of CYP induced a significant decrease in all the markers suggesting an anti-inflammatory effect of CYP (Table 4).

## Discussion

The number of people suffering from T2DM is increasing worldwide and has become a global public health problem. New treatment strategies are increasingly needed, and many studies have indicated that natural food constituents, such as resistance starch and bioactive compounds (e.g. phytochemicals), could be incorporated into a healthy balanced diet to aid in the prevention or management of T2DM.

Yam (*Dioscorea* spp.) is the fourth most important tuber crop after potatoes, cassava and sweet potatoes and contains a good source of essential dietary supplements such as protein, well-balanced essential amino acids and many dietary minerals^(44,45)^. Recently, interest has focused on yam as a potential insulin mimetic; thus, we searched the current literature to investigate whether yam and/or its extracts have the potential to help manage T2DM. We observed that the consumption of yam and/or its extracts had a beneficial effect on numerous glycaemic parameters including FBG, insulin, Hb1Ac and HOMA-IR. Additionally the consumption of yam and/or its extracts helped improvements in adiposity and circulating lipids, which are known to influence the development of T2DM.

However much of the work conducted on the effects of yam on T2DM has been shown in animal models and therefore further research is required in human participants, although ref. [46] has shown that the consumption of brown yam flour improved glycaemia in healthy subjects compared with other yam flours. In this review, we focused on studies conducted in models in which T2DM was induced by diet or in animals genetically predisposed to developing T2DM rather than those in which diabetes was induced by an injection of STZ, which is a model of type 1 diabetes. High fat feeding in mice leads to obesity, hyperinsulinaemia and altered glucose homoeostasis due to insufficient compensation by islets, thus modelling the human situation more accurately^(47)^, while STZ administration damages pancreatic *β*-cells depicting type 1 diabetes^(48)^. However, we also included rodent models in which T2DM develops spontaneously this included the OLEFT model, in which animals inherit diabetes, KK-A^y^ mice that develop obesity and severe hyperinsulinaemia^(49)^, again mimic human predisposition to diabetes. Furthermore, in these models, insulin sensitivity can be reversed via dietary manipulation and/or pharmacological administration as well as enable us to understand possible mechanisms^(49)^.

All papers identified in this review showed that the consumption of yam and/or its extracts at various doses improved glycaemia by improving fasting glucose levels and insulin sensitivity. As mentioned earlier, starch is the most abundant component of yam; cooking alters the properties of the starch making it more resistant to digestion. Resistant starch has been shown to prevent hyperglycaemia and reduce the risk of diabetes^(12,50,51)^ and lower serum TAG and LDL-cholesterol due to reduction in fat absorption^(52,53)^. However, all studies identified in this review used extracts to treat the rodents; thus, it is unlikely that an increase in resistance starch was responsible for the observed effects, but does highlight that the consumption of yam or its extracts has similar effects on glycaemia.

Another possible reason for the observed improvements in glycaemia maybe due to the inhibition of *α*-glucosidase; indeed yam and its extracts have been shown to be potent inhibitors of this enzyme^(27,54)^. *α*-glucosidase is located on the brush border of the small intestine and breaks down starch to glucose, and many *α*-glucosidase inhibitors, such as quercetin and acarbose, have been developed into clinical drugs to reduce blood glucose levels^(55–58)^. Not only do these inhibitors reduce FBG^(59)^ they also reduce post-prandial hyperglycaemia, thus reducing Hb1Ac^(60)^. However, only two of the eight studies measured this, but both found significant decreases^(35,37)^. These inhibitors can also influence the release of the incretin glucagon-like peptide 1, in support ref. [23] showed that allantoin (a yam extract) can increase glucagon-like peptide 1 release in a rat model of STZ induced diabetes. Indeed, we found that yam and/or its extracts treatment led to a decrease in plasma insulin in the three studies, which measured insulin levels^(35,38,42)^. Furthermore, these inhibitors can reduce lipid deposition and reduce adipocyte size and TAG and LDL^(23,57,58)^; indeed, we observed reductions in TAG, LDL, NEFA and TC in five of the ten studies^(35,36,38,40,41)^. Thus, further supporting the notion that the consumption of yam and/or its extracts results in the inhibition of *α*-glucosidase to exert its effects. However, the potency of inhibition of *α*-glucosidase by yam and/or its extracts is dependent on the solvent used for extraction and may be one of the reasons why there was difference in the lowest working dose observed in the studies, even when the same yam extract was utilised^(27,54)^. Indeed, ref. [35] used sodium carboxymethyl cellulose, while ref. [38] used saline to dissolve dioscin.

Other modes of action that yam and/or its extracts could improve glycaemic parameters are via the amelioration of oxidative and inflammatory responses. The consumption of HFD and high lipid profile levels can lead to the development of oxidative stress and systematic inflammation^(61)^, which results in decreased insulin sensitivity leading to hyperinsulinaemia and hyperglycaemia causing a pre-diabetic state. If uncontrolled, this can hinder the ability of the *β*-cells to meet demand leading to the development of diabetes^(62)^. This further exacerbates oxidative stress and inflammation leading to complications such as hypertension^(63)^. Numerous studies support the notion that yam and/or its extracts have antioxidant and anti-inflammatory properties^(64–66)^.

Indeed, we found that treatment with CYP was found to decrease pro-inflammatory markers NF-κB, MMP-3, IL-1B and anti-inflammatory marker IL-10^(36)^. IL-10 increases with obesity to protect against the disruption of insulin signalling; thus, ref. [36] concluded that CYP acted to reduce pro-inflammatory cytokines rather than stimulating anti-inflammatory cytokines. Extracts from *D. batatas* decrease the expression of pro-inflammatory cytokines TNF-*α*, monocyte chemoattractant protein-1 (MCP-1) and IL-6 in obese rodents^(67)^. In addition, the levels of PPARγ coactivator 1*α* (PGC-1*α*) in the pancreas return to basal expression levels in those animals of normal weight, similar to the effects of the drug metformin^(68)^. PGC-1*α* deficiency in the pancreas leads to an increase in pro-inflammatory markers production via NF-*κ*B, which in turn can lead to further damage of the pancreatic tissue^(69)^.

In support of the antioxidant properties, we found a decrease in Hb1Ac in two of the eight studies^(35,70)^. Hb1Ac is a known marker associated with increased oxidative stress^(71)^. In addition, we observed in one study that treatment with dioscorin resulted in a decrease in SBP possibly via angiotensin converting enzyme and vasorelaxation^(39,70)^. Many studies have implicated oxidative stress in hypertension, as reactive oxygen species influence vascular, renal and cardiac function and structure^(72)^. Further evidence of antioxidant/anti-inflammatory properties arises for the fact that yam and/or its extracts can restore the activity of the phosphoinositide 3 kinase/protein kinase B (Akt) and PPARγ pathways, both known to be suppressed in diseases associated with oxidative stress and inflammation^(38)^. Additionally, ref. [73] reported that *Sanggua Drink* extract, which consists of *Dioscorea*, might alleviate insulin resistance in HFD fed rodents via the induction of the PI3K/Akt signalling pathway.

The antioxidant/inflammatory properties of yam and/or its extracts may be due to the high phenolic and flavonoid content; these phenolic and flavonoids can reduce reactive oxygen species and reactive nitrogen species protecting pancreatic *β*-cells disruption^(74,75)^. Indeed, dioscorin has both dehydroascorbate reductase and monodehydroascorbate reductase activity enabling the generation of ascorbate which in turn to reduce the levels of reactive oxygen species^(76)^, thus proposed to be a good reducing agent. Furthermore, in STZ treated mice both allantoin and diosgenin can increase superoxide dismutase activity and the levels of reduced glutathione suggesting a reduction in oxidative stress. Moreover, allantoin treatment in STZ rats has been shown to reduce *β*-cell granulation suggesting that yam may play a potential role in protecting *β*-cells function and preventing granulation.

Many of the studies focused on the effects of yam and/or its extract on diabetes have investigated the indirect mechanism of improving glycaemia as discussed above. However, evidence for whether yam and/or its extracts have direct effect on the pancreatic *β*-cells or other cell types is limited, despite some evidence of a possible direct mechanism. Pancreatic lipase is released from the pancreas causing a reduction in the absorption and digestion of dietary TAG. *D. opposite* has been shown to inhibit pancreatic lipase secretion to a similar level to orlistat (the only FDA approved drug that inhibits pancreatic lipase to prevent 30 % for fat absorption)^(77–79)^. This could be associated with changes in lipid profile observed in five of the studies^(35,36,38,40,41)^ and decrease in body weight observed by four of the studies^(35,36,38,40)^. Further evidence for direct effects on the pancreas is the ability of yam, particularly *D. batatas* and allantoin, to prevent the loss of pancreatic mass by protecting against loss of islets, structural damage and atrophy in STZ-HFD mice^(68,80)^; however, this effect could potentially be indirect via amelioration of inflammation and oxidative stress, thus warrants further studies. There is also evidence that yam and/or its extracts improve glycaemia via improvements in adipose and muscle tissue. Dioscin and *D. batatas* administration decreases visceral adipose tissues and lipid profile, although this is thought to be via the improvements in inflammation. Interestingly, there is evidence of direct effects on muscle, administration of allantoin improved glucose uptake in skeletal muscle isolated from STZ-diabetic rats, possibly via increased translocation of GLUT 4, leading to increased MCR as suggested by ref. [21,43]. Furthermore, administration of *D. batatas* or allantoin in STZ-diabetic rodents increased microfibre number and area. It is well known that insulin resistance is manifested by a decrease in insulin stimulate glucose uptake in skeletal muscle. Nonetheless, the mechanism by which yam and/or extracts influence this is unknown and warrants further investigation.

### Limitation

While all the studies identified in this review and others conducted in STZ rodents agree that the consumption of the various yam and/or its extracts improves diabetic outcomes, there are some limitations to the study. These include not being able to agree on one species of yam or extract, or specific dose or length of consumption. Furthermore, the data extracted from the studies were descriptive and not actual values; therefore, a meta-analysis was not possible. However, the biggest limitation of this study is the lack of human studies. These are required to determine whether the effects observed in animal studies can be translated and, moreover, allow us to assess whether the consumption of yam mimics those observed following the consumption of the extracts, to assess whether the process of cooking yam alters glycaemia and finally to understand whether lifestyle and habitual diet could influence the effects observed.

### Conclusion

In summary, yam and its extracts have the potential to act as functional foods in the treatment of T2DM in numerous ways. However, further studies are required to understand potential mechanisms particularly in understanding the molecular pathways associated with insulin and glucose in the various important tissues (pancreas, muscle, adipose tissue) and to understand whether these are direct or indirect. Furthermore, human studies are required alongside studies comparing yam to similar to reliably inform dietetic practice, guidelines and policy makers.

## Supplementary material

For supplementary material accompanying this paper visit http://doi.org/10.1017/S0007114521003706. click here to view supplementary material
