# Supplementary material for: Could consumption of yam (Dioscorea) or its extract be beneficial in controlling glycaemia: a systematic review
Source: Br J Nutr. 2021 Sep 15;128(4):613–24. doi: 10.1017/S0007114521003706 (PMC9346617; doi:10.1017/S0007114521003706)
Supplement: Supplementary file 1 [file S0007114521003706sup001.docx]

*Supplementary Appendices*

**Appendix A: Quality Assessment**

|  | Cheng et al. 2019 | | Li et al. 2019 | | Moon et al. 2014 | | Sato et al. 2016 | | Shih et al. 2015 | | Wu et al. 2018 | |
| --- | --- | --- | --- | --- | --- | --- | --- | --- | --- | --- | --- | --- |
| Bias | Judge-ment | Support for judgement | Judge-ment | Support for judgement | Judge-ment | Support for judgement | Judge-ment | Support for judgement | Judge-ment | Support for judgement | Judge-ment | Support for judgement |
| Random sequence generation (selection bias) | Unclear risk of bias | Random assignmentno detail of sequence process | Unclear risk of bias | Random assignment no detail of sequence generation process used | High risk of bias | No mention of random component or sequence generation | Unclear risk of bias | Random assignment to group, no detail of sequence generation process used | Unclear risk of bias | Random assignment to group, no detail of generation process used | Unclear risk of bias | Random assignment to group, no detail of generation process used |
| Baseline characteristics*  (selection bias) | Low risk of bias | Characteris-tics were adequately described | Low risk of bias | Characteris-tics were adequately described | Unclear risk of bias | Characteris-tics similar at baseline, no description of disease induction | Unclear risk of bias | Genetic disease induction, insufficient description of baseline characteris-tics | Low risk of bias | Characteris-tics were adequately described | Low risk of bias | Characteris-tics were adequately described |
| Allocation concealment (selection bias) | Unclear risk of bias | No information on allocation conceal-ment | Unclear risk of bias | No information on allocation conceal-ment | Unclear risk of bias | No information on allocation conceal-ment | Unclear risk of bias | No information on allocation conceal-ment | Unclear risk of bias | No information on allocation conceal-ment | Unclear risk of bias | No information on allocation conceal-ment |

| Random housing (perform-ance bias) | Low risk of bias | Conditions controlled for all rodents (i.e. temperature, cage conditions) | Low risk of bias | Conditions controlled for all rodents (i.e. temperature, cage conditions) | Unclear risk of bias | Information on the housing lacks description on room temperature and cage conditions | Unclear risk of bias | Controlled conditions, lack of detail on temperature and cage conditions | Low risk of bias | Conditions controlled for all rodents (i.e. temperature, cage conditions) | Low risk of bias | Conditions controlled for all rodents (i.e. tempera-ture, cage conditions) |
| --- | --- | --- | --- | --- | --- | --- | --- | --- | --- | --- | --- | --- |
| Blinding (performance bias) | High risk of bias | No information on blinding of caretakers | High risk of bias | No information on blinding of caretakers | High risk of bias | No information on blinding of caretakers | High risk of bias | No information on blinding of caretakers | High risk of bias | No information on blinding of caretakers | High risk of bias | No information on blinding of caretakers |
| Random outcome assess-ment (detection bias) | Low risk of bias | 8 of 14 from each group were randomly selected | Low risk of bias | All animals were used for outcome assessment | Unclear risk of bias | No description of how the animals were selected | Low risk of bias | All animals were used for outcome assessment | Unclear risk of bias | No description of how the animals were selected | Un-clear risk of bias | No description of how the animals were selected |
| Blinding (detection bias) | High risk of bias | No information on blinding investigators | High risk of bias | No information on blinding investigators | High risk of bias | No information of blinding investigators | High risk of bias | No information of blinding investigators | High risk of bias | No information of blinding investigators | High risk of bias | No information of blinding investigators |
| In-complete outcome data (attrition bias) | High risk of bias | Only 8 per group were used for outcome measures | Low risk of bias | Data was collected from all rodents | Unclear risk of bias | No description of how many rodents were used for outcome assessment | Low risk of bias | Data was collected from all rodents | Unclear risk of bias | No description of how many rodents were used for outcome assessment | Un-clear risk of bias | No description of how many rodents were used for outcome assessment |
| Selective outcome reporting (reporting bias) | Unclear risk of bias | Protocol was not described in the manuscript, all expected outcomes described | Unclear risk of bias | Protocol was not described in the manuscript, all expected outcomes described | Unclear risk of bias | Protocol was not described in the manuscript, all expected outcomes described | Unclear risk of bias | Protocol was not described in the manuscript, all expected outcomes described | Unclear risk of bias | Protocol was not described in the manuscript, all expected outcomes described | Un-clear risk of bias | Protocol was not described in the manuscript, all expected outcomes described |
| Other bias | Low risk of bias | No other issues that could result in high risk of bias | Low risk of bias | No other issues that could result in high risk of bias | Unclear risk of bias | Unclear on influence of funding source | Unclear risk of bias | Unclear on influence of funding source | Unclear risk of bias | Unclear on influence of funding source | Un-clear risk of bias | Unclear on influence of funding source |

|  | Hashidume et al. 2018 | | Hsu et al. 2007 | | Kim et al. 2012 | | Xu et al. 2020 | |
| --- | --- | --- | --- | --- | --- | --- | --- | --- |
| Bias | Judgement | Support for judgement | Judgement | Support for judgement | Judgement | Support for judgement | Judgement | Support for judgement |
| Random sequence generation (selection bias) | Unclear risk of bias | Random assignment by bodyweight into groups, no detail of generation process used | Unclear risk of bias | Random assignment, no detail of sequence generation process used | Unclear risk of bias | Random assignment into groups, no detail of generation process used | Unclear risk of bias | Indication of random group assignment throughout, no detail of random sequence generation |
| Baseline characteristics*  (selection bias) | Low risk of bias | Characteristics were adequately described | Low risk of bias | Characteristics were adequately described | Low risk of bias | Characteristics were adequately described | Low risk of bias | Characteristics were adequately described |
| Allocation concealment (selection bias) | Unclear risk of bias | No information on allocation concealment | Unclear risk of bias | No information on allocation concealment | Unclear risk of bias | No information on allocation concealment | Unclear risk of bias | No information on allocation concealment |
| Random housing (performance bias) | Low risk of bias | Conditions controlled for all rodents (i.e. temperature, cage conditions) | Low risk of bias | Conditions controlled for all rodents (i.e. temperature, cage conditions) | Low risk of bias | Conditions controlled for all rodents (i.e. temperature, cage conditions) | Low risk of bias | Conditions controlled for all rodents (i.e. temperature, cage conditions) |
| Blinding (performance bias) | High risk of bias | No information on blinding of caretakers | High risk of bias | No information on blinding of caretakers | High risk of bias | No information on blinding of caretakers | High risk of bias | No information on blinding of caretakers |
| Random outcome assessment (detection bias) | Unclear risk of bias | Used all animals for most outcomes (n=7 per group) however some outcomes only assessed 4 or 5 without mention of random selection | Unclear risk of bias | No mention of random selection from each group | Unclear risk of bias | Used all animals for most outcomes (n=8 per group) however GLUT4 translocation data only assessed 5-6 per group without mention of random selection | Unclear risk of bias | Mice and rat groups consisted of n=10, only 8 were used for outcome assessments, no mention of random selection |
| Blinding (detection bias) | High risk of bias | No information of blinding of investigators | High risk of bias | No information of blinding of investigators | High risk of bias | No information of blinding of investigators | High risk of bias | No information of blinding of investigators |

| Incomplete outcome data (attrition bias) | Unclear risk of bias | Used all animals for most outcomes (n=7 per group) however some outcomes only assessed 4 or 5 with no explanation why all animals were not used | Unclear risk of bias | Only 8 per group were used for outcome measures (some groups contained 10 or 12 animals) with no explanation why all animals were not used | Unclear risk of bias | Used all animals for most outcomes (n=8 per group) however GLUT4 translocation data only assessed 5-6 per group without an explanation why all animals were not used | High risk of bias | Mice and rat groups consisted of n=10, only 8 were used for outcome assessments with no explanation why all animals were not used |
| --- | --- | --- | --- | --- | --- | --- | --- | --- |
| Selective outcome reporting (reporting bias) | Unclear risk of bias | Protocol was not described in the manuscript, all expected outcomes described | Unclear risk of bias | Protocol was not described in the manuscript, all expected outcomes described | Unclear risk of bias | Protocol was not described in the manuscript, all expected outcomes described | Unclear risk of bias | Protocol was not described in the manuscript, all expected outcomes described |
| Other bias | Unclear risk of bias | Unclear on influence of funding source | Unclear risk of bias | Unclear on influence of funding source | Unclear risk of bias | Unclear on influence of funding source | Unclear risk of bias | Unclear on influence of funding source |
